# The Effect of RANKL/OPG Balance on Reducing Implant Complications

**DOI:** 10.3390/jfb8040042

**Published:** 2017-09-22

**Authors:** Elizabeth R. Kapasa, Peter V. Giannoudis, Xiaodong Jia, Paul V. Hatton, Xuebin B. Yang

**Affiliations:** 1 Doctoral Training Centre—Regenerative Medicine, Institute of Medical and Biological Engineering, School of Mechanical Engineering, University of Leeds, Leeds LS2 9JT, UK; mnerk@leeds.ac.uk; 2 Biomaterials and Tissue Engineering Group, School of Dentistry, University of Leeds, Leeds LS2 9JT, UK; 3 Department of Trauma and Orthopaedic Surgery, School of Medicine, University of Leeds, Leeds LS2 9JT, UK; P.Giannoudis@leeds.ac.uk; 4 School of Chemical and Process Engineering, University of Leeds, Leeds LS2 9JT, UK; X.Jia@leeds.ac.uk; 5 School of Clinical Dentistry, University of Sheffield, Sheffield S10 2TA, UK; P.V.Hatton@sheffield.ac.uk

**Keywords:** RANKL, OPG, implants, complications, bone loss, bone, bone regeneration

## Abstract

Despite the phenomenal success of implants particularly in the realms of dentistry and orthopaedics, there are still challenges to overcome. The failure of implants resulting from infection, prosthetic loosening, and non-union continue to be the most notorious examples. The cascade of fracture healing and bone repair, especially with the presence of an implant, is complex because it involves a multifaceted immune response alongside the intricate process of bone formation and remodelling. Bone loss is a serious clinical problem that is frequently accompanied by chronic inflammation, illustrating that there is a convoluted relationship between inflammation and bone erosion. The effects of pro-inflammatory factors play a significant role in initiating and maintaining osteoclastogenesis that results in bone resorption by osteoclasts. This is because there is a disruption of the relative ratio between Receptor Activator of Nuclear Factor κB-Ligand (RANKL) and osteoprotegerin (OPG), which is central to modulating bone repair and remodelling. This review aims to provide a background to the bone remodelling process, the bone repair cascade post-implantation, and the associated complications. Furthermore, current clinical solutions that can influence bone formation via either internal or extrinsic mechanisms will be described. These efficacious treatments for osteolysis via targeting the RANKL/OPG ratio may be crucial to reducing the incidence of related implant failures in the future.

## 1. Introduction—The Clinical Need

Our growing ageing population suffers from chronic age-related diseases such as osteoporosis and rheumatoid arthritis (RA). Approximately 2% of the world population suffer from RA with chronically inflamed joints that deteriorates the bone and cartilage until the joint needs to be replaced [1]. Diseases such as RA affects approximately 1.3 million in the U.S. [2] and have increased the prevalence of total joint replacements. Although there was a report which stated that the incidence of orthopaedic surgery in patients with RA has been declining over the past two decades; however, in a UK study of RA patients, large-joint replacement rates remaining constant over time [3]. Hip replacements were among the most successful surgical procedure developed in the 20th century [4]. However, a proportion of these prostheses fail and require surgical revision due to aseptic loosening caused by inflamed peri-prosthetic tissue rich in macrophages, cytokines, and wear particles [4,5]. In dentistry, the prevailing ailment responsible for bone loss is periodontitis, which can lead to dental implants to replace the damaged tooth [1,6]. These orthopaedic implants replace the impaired bone to help restore normal function to patients, relieve suffering from pain, and subsequently improve their quality of life.

Despite the phenomenal success of implants particularly in the realms of dentistry and orthopaedics, there are still challenges to overcome. The failure of implants resulting from infection, prosthetic loosening, and non-union continue to be the most notorious examples [4]. Chronic systemic inflammation, being one of the hazards of derangement of the homeostatic mechanisms of the musculoskeletal system, can lead to serious bone loss, which illustrates that there is a convoluted relationship between inflammation and bone erosion [1]. Significantly elevated levels of Receptor Activator of Nuclear Factor Kappa-B Ligand (RANKL), Tumour Necrosis Factor-α (TNF-α), and Interleukin-1 (IL-1) are frequently observed in inflamed tissues adjacent to local osteolysis compared to relevant controls in RA, periodontitis, and peri-prosthetic tissues [1,4,6,7]. Although these debilitating and painful diseases are distinct from one another, they share the same key pro-inflammatory mediators, TNF-α and IL-1, that may lead to excessive bone resorption by the priming of osteoclasts [1,4,6]. 

## 2. The Roles of RANK, RANKL, and OPG in Bone Remodelling

The human skeleton consists of more than 200 bones that facilitates movement, protects vital organs, provides aesthetic features, and supplies a reservoir of blood cells and minerals. These key functions require a bone structure that, when exposed to different mechanical forces, can subsequently be remodelled to counteract normal wear and tear throughout life [8]. This remodelling process is regulated by molecular mechanisms that influence osteoblasts and osteoclasts, which respectively form new bone and resorb damaged/unneeded bone to achieve a healthy homeostatic balance. However, when this dynamic equilibrium is disrupted, it results in osteopathic conditions such as osteoporosis (decreased bone mass), osteopetrosis (increased bone mass), and Paget’s disease (increased bone remodelling) [8,9]. In the last decade, the discovery of the RANK/RANKL/OPG triad has been significant in aiding our understanding of the bone remodelling process and consequently the causes of bone diseases [8,10,11]. The critical role of these three molecules has been demonstrated using transgenic and gene-knockout studies, but there are aspects of bone biology that remain poorly understood [1,12].

**Figure 1:**
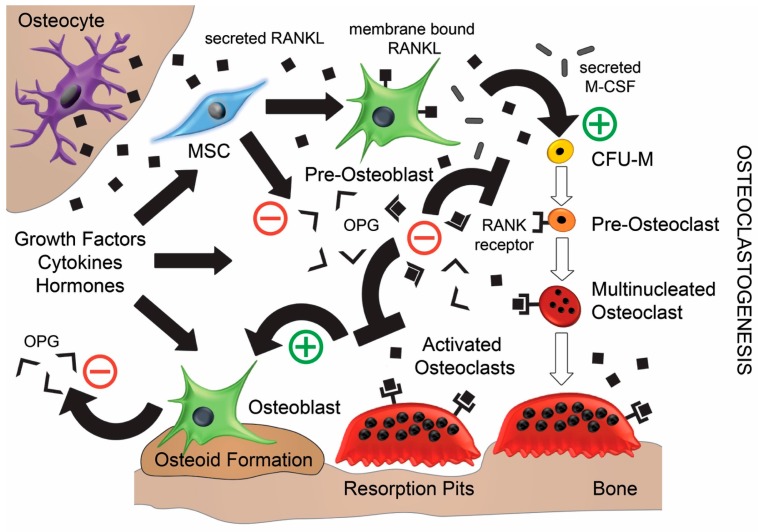
Mechanism of bone remodelling involving RANK/RANKL/OPG effects on osteoblasts and osteoclasts [11,19]. RANKL: Receptor Activator of Nuclear Factor κB-Ligand; OPG: osteoprotegerin; MSCs: mesenchymal stem or stromal cells; M-CSF: Macrophage Colony-Stimulating Factor; CFU-M: Colony Forming Unit-Macrophages.

In healthy bone, embedded osteocytes are central in orchestrating normal bone remodelling by modulating bone-forming osteoblasts and bone-resorbing osteoclasts through cell signalling molecules, most prolifically through RANKL (Figure 1) [13]. It is known that microfractures frequently occur with approximately 1–2 million sites in the adult skeleton that initiate the bone remodelling process in what is called a bone remodelling unit [8]. Osteocytes (mature osteoblasts embedded in bone matrix) are stellar-shaped with long processes that detect these microfractures and undergo apoptosis, which stops the release of sclerostin (osteogenesis inhibitor) [14,15,16]. Neighbouring osteocytes sense this and release biomolecular signals, including growth factors and cytokines that attract bone marrow-derived mesenchymal stem or stromal cells (MSCs). These MSCs proliferate and differentiate into pre-osteoblasts that secrete Macrophage Colony-Stimulating Factor (M-CSF) and express RANKL [17]. These stimulate colony-forming unit–macrophages (CFU-M, macrophage pre-cursors) to differentiate into pre-osteoclasts, which fuse into multinucleated osteoclasts that display RANK receptors. Therefore, inflammation is the initial phase needed in bone remodelling, as well as bone healing. 

Osteoclasts become activated when bound to RANKL either indirectly, in its soluble form or directly, to membrane-bound RANKL on the pre-osteoblasts [10,17]. Then, bone-lining cells expose the bone matrix surface to the osteoclasts that seal a zone where they secrete acid (e.g., hydrochloric acid) and hydrolytic enzymes (e.g., cathepsin K), which resorbs the damaged bone [8]. This simultaneously releases fragments of growth factors from the matrix including Transforming Growth Factor-β (TGF-β) and Bone Morphogenetic Proteins (BMPs), which stimulates the differentiation of MSCs into mature osteoblasts [16,18]. Then, the osteoblasts line the resorbed cavity and fill it with osteoid which forms woven bone. Synchronously, osteoblasts secrete osteoprotegerin (OPG) that acts as a decoy receptor, antagonistically binds to RANKL which reduces osteoclastogenesis and drives apoptosis of the pre-existing osteoclasts [12,18]. Finally, the woven bone is mineralised over a few months by the osteoblasts, which eventually undergo apoptosis, or become embedded and differentiate into osteocytes, or differentiate further into bone-lining cells [18]. 

A number of studies have shown that bone remodelling is dependent on the ratio of RANKL to OPG. If RANKL is higher, then bone resorption occurs; conversely, if OPG is higher, then bone formation dominates. Thus, OPG acquired its name from its ability to protect bone from excessive resorption by counteracting the osteoclastic effects of RANKL [8,9,12,20]. However, RANK, RANKL, and OPG are also expressed by other cells including fibroblasts, endothelial cells, and inflammatory cells, which enables them to modulate bone metabolism [6,10,21]. Imbalances of bone metabolism ensue from growth factors, cytokines, and hormones that disturb the RANKL/OPG ratio [11,22]. For example, parathyroid hormone stimulates bone formation at low concentrations, and contrarily induces bone resorption by promoting RANKL [11].

## 3. The Bone Repair Cascade Post-Implantation

The cascade of fracture healing and bone repair, especially with the presence of an implant, is complex because it involves a multifaceted immune response alongside the intricate process of bone formation and remodelling. Bone can heal through either primary (direct) or secondary (indirect) fracture healing; primary involves direct osteogenesis via bone remodelling units with little resorption and no callus formation, compared to secondary fracture healing which is more complex. Most fractures heal by secondary fracture healing, which consists of a combination of intramembranous ossification in the internal hard callus, and endochondral ossification in the external soft callus, in order to quickly bridge the gap, stabilise the fracture, and regain biomechanical function [16,23]. MSCs differentiate into osteoblasts and form bone in intramembranous ossification, in contrast to endochondral ossification that is comprised first of cartilage that is later calcified and transformed into bone. The overlapping phases of this repair process are controlled and regulated by signalling molecules that can be divided into three main groups: pro-inflammatory cytokines, growth factors including the TGF-β superfamily members, and angiogenic factors [24].

Inflammation is the primary phase that initiates normal bone turnover. Post-implantation, there is an immediate acute inflammatory response for the first three days. A haematoma is formed containing platelets and inflammatory cells that release Platelet-Derived Growth Factor (PDGF), TGF-β, and pro-inflammatory cytokines, including TNF-α, IL-1, and IL-6, that recruit other inflammatory cells and MSCs [23]. These pro-inflammatory cytokines increase expression in T-cells and osteoblasts for RANKL and M-CSF, whilst OPG expression is reduced, in order to cultivate osteoclasts to resorb necrotic tissue [6,21,25]. Migrated MSCs are stimulated by the TGF-β superfamily members, growth differentiation factors (GDFs), and BMPs to proliferate and differentiate into osteogenic and chondrogenic lineages, respectively, to begin intramembranous and endochondral (via cartilage) ossification [26]. During the cartilage formation phase, OPG expression in chondrocytes peaks, leading to an increase in bone mass through the negative regulation of osteoclast formation; then the OPG expression decreases as calcified cartilage is substituted by woven bone [25,27]. RANKL, M-CSF, and TNF-α are considered to aid migration of bone cells to start cartilage mineralisation [26]. After about 14 days, these cells cease to proliferate and express Vascular Endothelial Growth Factor (VEGF) to encourage neoangiogenesis by endothelial MSCs to form vascularised woven bone [23,26]. Subsequently, there is another rise in IL-1, IL-6, and TNF-α respectively, which upregulates RANKL in osteoblasts and drives osteoclastogenesis [10,24]. In particular, IL-β1 can stimulate pre-osteoclasts directly to fuse into multinucleated osteoclasts and can inhibit apoptosis to prolong their lifespan [28]. Therefore, osteoclasts can resorb the woven bone, which is then remodelled into lamellar cortical bone and trabecular bone by osteoblasts as previously described.

## 4. Post-Implantation Complications

The local damage caused by implantation and the presence of a foreign material elicits an immune response that uses chemokines to attract circulating monocytes to the area, which differentiate into activated macrophages that release TNF-α, IL-1, IL-6, and M-CSF [28,29]. In chronic inflammation, these macrophages exist in abundance and have been found to differentiate further into pre-osteoclasts and then into mature osteoclasts in the presence of M-CSF [1]. Furthermore, these macrophages can express RANK, RANKL, and M-CSF, which enables self-activated osteoclastogenesis. The process is accelerated when macrophages ingest prosthetic wear particles [4,29,30,31,32,33]. The use of bone cements to fix implants has also been shown to increase RANKL production and therefore the RANKL/OPG ratio [34,35]. In addition, T-cells contribute to the release of RANKL, M-CSF, TNF-α, and IL-1, which can trigger pre-osteoblasts and MSCs to also express RANKL, thus further increasing the RANKL/OPG ratio [21,29,36,37]. Moreover, prostaglandin-E_2_ and 1,25(OH)_2_D_3_ (active metabolite of vitamin D) were often observed in peri-prosthetic tissue and found to contribute to increasing the RANKL/OPG ratio [38]. Other researchers have demonstrated that TNF-α and IL-1 can induce osteoclastogenesis alone in the absence of RANKL [28,39]. Finally, the accompaniment of IL-6 expression from inflammatory cells has been correlated with osteoclast formation and peri-prosthetic infection [40,41]. In summary, surrounding the implant there is a large host of macrophages that in the presence of M-CSF, can differentiate to create a large pool of active osteoclasts via two pathways: by binding to RANKL secreted from the various cells aforementioned; or by direct stimulation from TNF-α and IL-1. 

**Figure 2:**
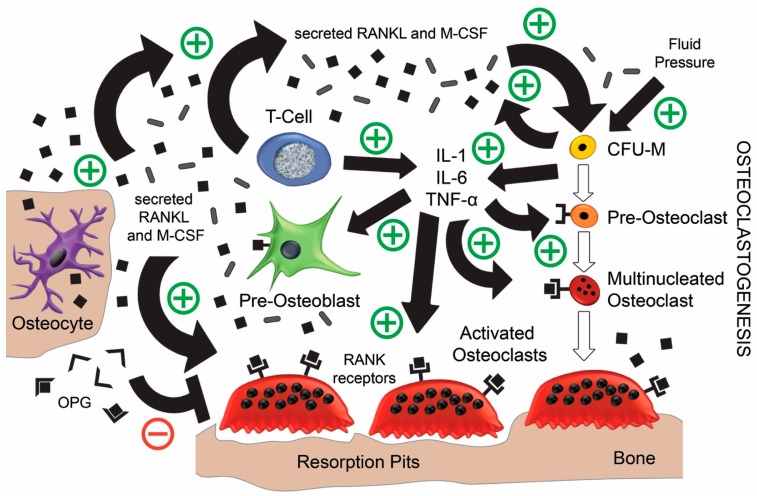
The amassed adverse chronic effects of inflammatory cytokines and chemokines, which results in osteoclastogenesis and osteolysis that supersedes the negative feedback by OPG [1,11,19,21,28]. RANKL: Receptor Activator of Nuclear Factor κB-Ligand; OPG: osteoprotegerin; M-CSF: Macrophage Colony-Stimulating Factor; CFU-M: Colony Forming Unit–Macrophages; TNF-α: Tumour Necrosis Factor-α; IL-1: Interleukin-1; IL-6: Interleukin-6.

It is worth noting that macrophages isolated from peri-prosthetic tissue have been found to differentiate into mature osteoclasts without the company of MSCs or osteoblastic cells, and contained higher levels of RANKL mRNA compared to OPG mRNA [29]. In fact, most of the RANKL is produced by leucocytes, which negates the need for osteoblastic cells or MSCs for support [1]. A number of studies have noted high RANKL/OPG ratios compared to controls, and the level of severity was correlated with higher ratios across different bone loss pathologies [6,28,42]. These results indicate that there is markedly more RANKL readily available to bind with RANK than there is OPG to counteract its effect, so the activity of OPG is dampened. Furthermore, there is the natural generation of bone resorption exerted by mechanical stimulation and fluid pressure which is significantly enhanced when exposed to osteolytic cytokines that assists the progression of aseptic loosening [43,44,45]. All of these findings demonstrate this dire situation illustrated in Figure 2, that chronic inflammation rapidly cascades into severe osteolysis which inevitably requires clinical intervention [1,11,21,28].

## 5. Current Clinical Solutions to Correct the RANKL/OPG Balance

There has been a significant research effort to try to manipulate implant surface topography and chemistry to accelerate and enhance bone healing [46,47,48]. Research has shifted from being bioinert to producing bioactive properties including osteoinduction (directing differentiation down an osteoblastic lineage), osteoconduction (producing bone growth), and osseointegration (supporting bone ingrowth to unify the material with the surrounding bone) [48,49]. Implants have also been designed with pores and incorporated with antibiotics or growth factors, such as BMP-2 and VEGF, to encourage bone tissue ingrowth and angiogenesis in order to prevent osteolysis developing post-implantation.

A number of studies have shown that therapeutic interventions can be designed to address the bone remodelling by altering the RANKL/OPG ratio. This can be done through solutions that aim to promote bone formation either: (1) by internally correcting the balance of intrinsic RANKL/OPG expression, or (2) by introducing exogenous molecules to manipulate and enhance the RANKL/OPG ratio. 

### 5.1. Correcting the Intrinsic RANKL/OPG Balance Internally

For efficacious bone repair, the site must have an adequate blood supply and withstand a steady increase in biomechanical stability, and the implant should integrate into the native bone tissue [26]. Research has established that complications arise from metal implant corrosion in the body, leading to the release of metal ions that cause an inflammatory and immune response [50]. Implants are typically made from titanium or stainless steel. It is now known that osteoclasts can directly corrode titanium and stainless steel surfaces and then release ions, which induces the secretion of pro-inflammatory cytokines and RANKL, resulting in osteoclastogenesis and subsequent adjacent osteolysis and aseptic loosening [51,52,53]. Therefore, the choice of material may be crucial to altering RANKL/OPG ratios since studies conducted on failed implants have revealed differences in osteoclast formation depending on the material chemistry [29]. For instance, biosilica may be a possible prophylactic material that can upregulate OPG synthesis to support bone formation by decreasing the RANKL/OPG ratio [54,55]. Furthermore, microstructured titanium surfaces have been shown to enhance osseointegration by increasing OPG expression in bone remodelling [56]. Titanium nitride oxide coating on rough titanium surfaces has also been shown to stimulate the proliferation of human primary osteoblasts [57].

### 5.2. Enhancing the RANKL/OPG Ratio Extrinsically

Anti-inflammatory drugs obstruct chronic inflammation from developing by inhibiting the pro-inflammatory cytokines. For example, oral pentoxifylline has been successful in reducing TNF-α release from macrophages exposed to prosthetic particle debris [58]. This reduces osteoclast activity that is stimulated through TNF-α. However, anti-inflammatory drugs are only partially effective because they do not block the expression of RANKL, unlike OPG that can antagonise RANKL [21]. A multifaceted approach that targets TNF-α, IL-1, and RANKL may be more efficacious [1]. Today’s most popular anti-resorptive pharmaceutical are bisphosphonates that treat osteolytic conditions including Paget’s disease, osteoporosis, and bone metastases, by inhibiting osteoclast formation and promoting osteoblast maturation [59,60,61]. Bisphosphonates act by binding to bone minerals that are ingested by osteoclasts and disrupt their resorptive ability [60]. Although it has been debated whether this action can be sustained over long-term treatment, which has been associated with increased osteoclast populations [60].

On the other hand, OPG and OPG-like molecules are promising candidates to block RANK/RANKL activation and inhibit osteoclastogenesis in order to prevent or delay osteolysis and subsequent prosthetic failure [29]. It is clear that OPG is essential for maintaining a healthy bone metabolism by diminishing bone resorption and increasing the density, area, and strength of bone [11,62]. Genetic deletion of OPG in mice results in severe early onset osteoporosis and a high fracture incidence [9,10]. Therefore, it is unsurprising that therapeutic administration of OPG is successful in treating osteolytic conditions by supressing bone resorption [9,11,63].

The hallmarks of OPG have led to the development of denosumab, a fully human monoclonal antibody to RANKL that behaves like OPG by binding to RANKL and preventing subsequent osteoclastogenesis, but with the additional advantage of having a significantly longer half-life and therefore requiring less frequent administration [11,60]. Furthermore, current clinical results have demonstrated that denosumab is more efficacious than bisphosphonate treatments by rapidly reducing bone resorption to a greater extent, increasing overall bone mineral density, and significantly reducing fracture incidence [60,64]. This may be because denosumab inhibits RANKL, which is readily available in the extracellular space, and therefore is not limited like the bisphosphonates that rely on being endocytosed by osteoclasts to yield their actions internally [60]. Denosumab has been clinically successful in treating osteoporosis, rheumatoid arthritis, and bone metastases [65]. Therefore, denosumab could be modified in the future and used to treat other osteolytic pathologies, including excessive bone resorption post-implantation.

Conversely, bisphosphonates, denosumab, and any therapeutic that alters the RANKL/OPG ratio must be used with caution and closely monitored since a lack of normal, healthy bone remodelling can lead to other problems such as osteonecrosis of the jaw [66,67,68]. Bisphosphonates carry undesired side effects due to its non-specificity. In contrast, although the denosumab antibody is highly specific, it can travel to impact bone anywhere in the body [60]. Therefore, a conducive research effort should be made to adapt implants to include a limited local delivery of anti-RANKL therapeutics that would reduce chronic inflammation of the wound site, but concurrently allow acute inflammation that initiates the cascade for normal bone healing and remodelling to reduce the need for revision surgery. Aforementioned, to date this has been mainly achieved through altering intrinsic RANKL/OPG ratio internally by using implant surface topography and chemistry to influence cell behaviour to promote bone healing and reduce chronic inflammation.

## 6. Conclusions

Over the last decade, increased understanding of the complex aetiology of diseases related to bone metabolism has inspired the development of innovative, efficacious treatments for osteolysis that target the RANKL-L/OPG ratio to reduce the incidence of related implant failures. While mechanical properties of biomaterials remain important, it is recommended that future implant designs should give an even greater consideration to local cellular responses. Adoption of this more holistic approach has the potential to increase the rate of bone tissue regeneration, providing enhanced osseointegration and improved long-term clinical success.
